# Reply to Ju et al.: Mechanisms of deeper soil organic carbon loss

**DOI:** 10.1073/pnas.2510690122

**Published:** 2025-06-13

**Authors:** Zhenghu Zhoua, Minggang Xu, Andong Cai

**Affiliations:** ^a^Institute of Carbon Neutrality, School of Ecology, Northeast Forestry University, Harbin 150040, China; ^b^Key Laboratory of Sustainable Forest Ecosystem Management-Ministry of Education, Northeast Forestry University, Harbin 150040, China; ^c^State Key Laboratory of Efficient Utilization of Arid and Semi- arid Arable Land in Northern China, Institute of Agricultural Resources and Regional Planning, Chinese Academy of Agricultural Sciences, Beijing 100081, China; ^d^Institute of Environment and Sustainable Development in Agriculture, Chinese Academy of Agricultural Sciences, Beijing 100081, China

**Fig. 1.**
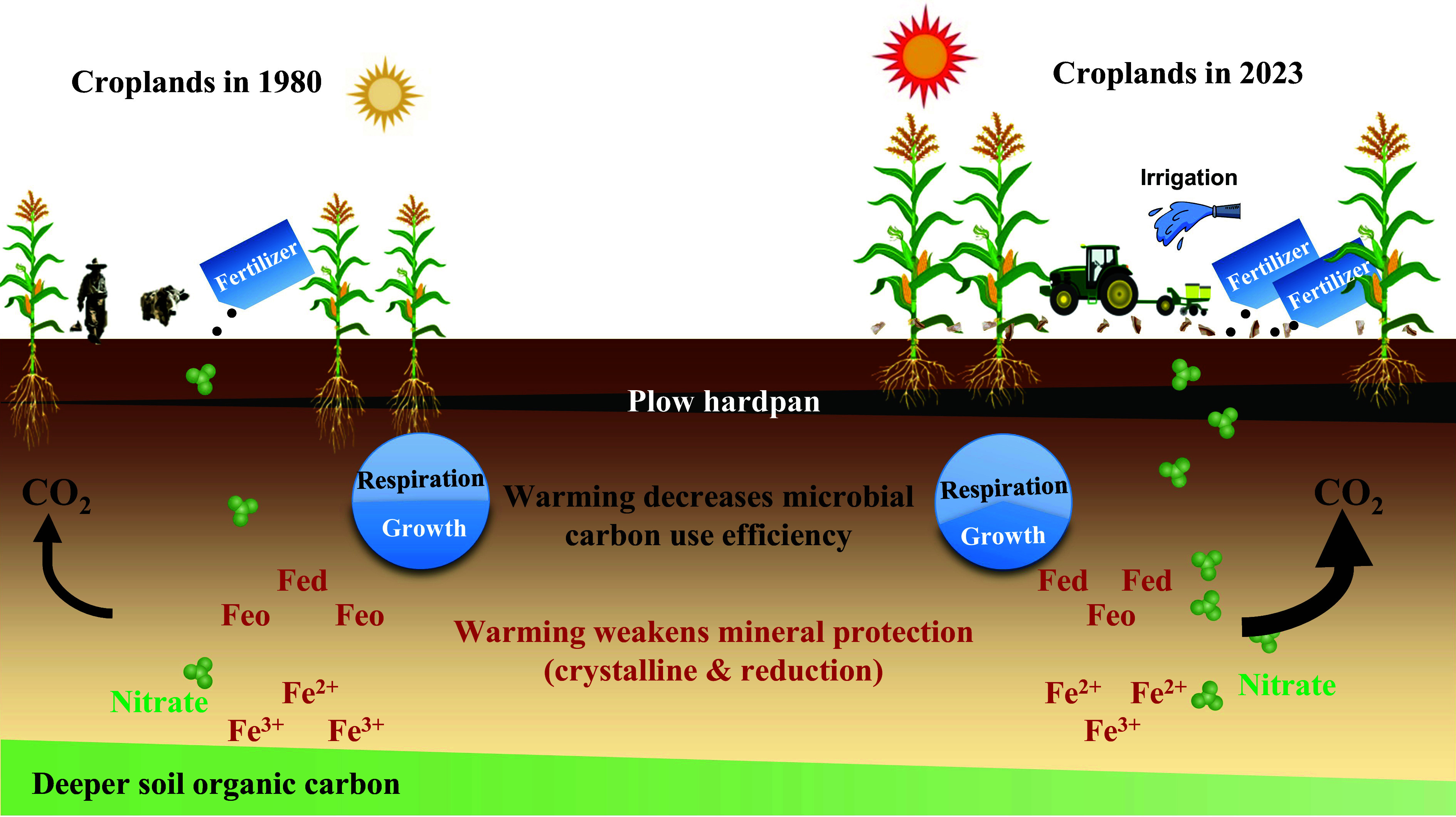
Mechanisms of deeper SOC loss.

In the accompanying comment, Ju et al. (1) supports the deeper soil organic carbon (SOC) loss from our continental-scale soil resampling study (2). Importantly, we greatly appreciate their novel mechanisms explaining the loss of deeper SOC based on a long-term (15-y) field fertilization experiment (1). In specific, O_2_ is in short supply in deeper soils, instead, nitrate serves as an electron acceptor for deeper soil microbial respiration under anoxic conditions. Therefore, it is expected that fertilization-induced nitrate accumulation in deeper soil would trigger the decomposition rate and result in the loss of SOC. Additionally, this process may be further amplified by climate warming through increased nitrate mobility via intensified hydrological leaching (1, 3). Besides the nitrate accumulation-induced decomposition proposed by Ju et al. (1) and the four potential mechanisms proposed by our original study (2), here, combining literature synthesis, we provide another two potential mechanisms (Fig. 1).

First, warming could promote deeper SOC decomposition by weakening mineral protection. This mechanism was supported by warming-induced decreases in reactive iron and aluminum concentrations (4), the accumulation of thermodynamically stable and highly crystalline minerals (*SI Appendix*, Fig. S1), and enhanced microbial Fe(III) reduction (5). In addition, temperature alters the adsorption–desorption equilibrium of dissolved organic carbon by soil minerals, while increasing temperature favors the desorption of SOC from soil minerals and destroys the stable organo-mineral associations (6).

Second, contrasting microbial physiological properties in top versus deeper soils may also contribute to the observed SOC loss. The per unit SOC in deeper soils supports a higher microbial biomass than in topsoil, making it more likely that warming-induced stimulation of microbial activity in deeper soils will lead to increased decomposition of deeper SOC (7). In addition, microbial carbon use efficiency was suggested to improve SOC storage (8). However, microbial communities in deeper soil have significant lower carbon use efficiency than that in topsoil (*SI Appendix*, Fig. S1); meanwhile, temperature had negative effects on microbial carbon use efficiency (8). Overall, agricultural management-induced increases in carbon inputs is difficult to reach deeper soils, consequently, warming-induced weakening mineral protection and shifts in microbial physiology would lead to deeper SOC loss at long-term scale.

We thank Ju et al. (1) for their constructive engagement, which enriches the narrative around subsoil SOC loss. Addressing subsoil carbon loss requires multiple approaches that simultaneously consider warming impacts, agricultural management, carbon input strategies, microbial physiology, and mineral protection. In addition, the study of deeper SOC dynamics is difficult because these SOC had significantly longer turnover time than topsoil SOC. We acknowledge that our large-scale resampling of whole-soil profiles SOC rang an alarm bell for deeper SOC loss but lacking directly evidence associated with above mechanism. Therefore, on the one hand, future study is urgently needed to clarify the mechanisms of the vulnerability of deeper SOC under climate change and agricultural management. On the other hand, effective efforts should be account for the protection of deeper SOC to achieving the carbon neutral targets, such as “4 per mille initiative” (9).
